# Supplementary material for: Reusable rule-based cell cycle model explains compartment-resolved dynamics of 16 observables in RPE-1 cells
Source: PLoS Comput Biol. 2024 Jan 8;20(1):e1011151. doi: 10.1371/journal.pcbi.1011151 (PMC10773963; doi:10.1371/journal.pcbi.1011151)
Supplement: Attachment — Submitted filename: response_to_reviewers_cell_cycle_plos.pdf [file pcbi.1011151.s002.pdf]

October 9, 2023

Paul F Lang
Email: plang@biosim.ai

Frohburgstrasse 106
8057 Zurich
Switzerland

Dear Dr Chen and Dr Lauffenberger,

Thank you very much for giving us the opportunity to revise our manuscript. We also apologize for the delay in our response. We are truly grateful for the thorough and insightful questions raised by the reviewers. They have not only guided us in improving weak spots in the manuscript and the clarity of our explanations, but have also prompted us to think about our research from fresh perspectives.

In the following, you will find our detailed responses to each of the reviewers' comments. We have also included the revised manuscript and supplementary materials, both with and without highlighted changes, for your convenience.

Sincerely yours,

Paul F Lang

Reviewer 1

> The authors construct a rule-based model of the DNA damage checkpoints in G1 and G2, successfully capturing known dynamics of cyclins E, A, and B. They also note that CDKN1A does not fully return to baseline prior to cell cycle reentry, consistent with a previous report by Stallaert et al. However, in this same paper, the authors demonstrate that proliferative effectors such as cyclin D and CDK4 increase with CDKN1A during cell cycle arrest, while total RB decreases, and that these changes may allow cells to reenter the cell cycle in the presence of residual CDKN1A. Does the model also capture these dynamics when TP53 is turned off? If so, can the authors provide any additional mechanistic insight into how cell cycle reentry is governed following DNA damage?

Thank you for bringing this up. Unfortunately, our model cannot capture these dynamics, as it assumes constant concentrations of cyclin dependent kinases (CDKs), RB, and cyclin D. After removing arrested cells, our analysis in the cell_cycle_time_course GitHub repository indicates that these assumptions approximately hold in proliferating cells. While our model indeed shows that the proliferative effector cyclin E is elevated upon reentry, we can therefore not reason about CDKs, RB and cyclin D levels, based on our model. However, we are aware that Cheng *et al.* EMBO J 18, 1571 (1991), observed that primary mouse embryonic fibroblasts lacking genes that encode CDKN1A and B express cyclin D at much reduced levels. This is also in agreement with the findings of Stallaert *et al*.

> One important regulatory event that uses effector compartmentalization to govern state cell cycle state transitions is the spatial positive feedback at the onset of mitosis described by Santos et al. (PMID: 22726437). Does the current model also capture this mechanism?

While our model accounts for CDK1 phosphorylation, it does not account for the phosphorylation of cyclin B itself. The current model version can therefore not describe the spatial positive feedback observed by Santos *et al.*, which would likely increase the robustness and predictive power of the model when simulating experimental perturbations.

> In a follow-up to the manuscript providing the experimental data supporting this manuscript (Stallaert et al., Cell Systems 2022), similar single-cell measurements of cell cycle effectors in response to perturbations, including DNA damage (Stallaert et al, MSB 2022, PMID: 36161508). I do not believe that modeling these new data is required in this manuscript; however, this seems like an obvious follow-up to this study to further test the model, and could be cited in the discussion when describing future steps.

Thank you very much for pointing us to this paper, which describes perturbation experiments including hypomitogenic stress (serum starvation), replication stress (etoposide), and oxidative stress (H_2O_2). We see two major challenges to using this data. First, our time course reconstruction procedure assumes asynchronously proliferating cells to map from an ordering of cells to cell cycle time (this is because distances in state space do not necessarily reflect distances in time). However, the perturbations in the study were too strong to be permissive for continued proliferation. Second, the chemical perturbations have more complex effects than single gene knockdowns, so one cannot be certain about how to simulate the conducted perturbation in the model. Third, the mechanisms leading to cellular senescence, polyploidy, endoreduplication, and mitotic skipping that are triggered by the tested perturbations are not included in our cell cycle model. Nevertheless, one can try to model the serum starvation condition with reduced cyclin D levels, and the H_2O_2 condition with elevated TP53, both of which are actually observed in the study. Assuming that the above mechanisms do not kick in immediately, one may try to fit the model to the data from early stages after perturbation. Overall, despite several complications, we do see potential avenues how this data could be utilized and decided to mention the paper in our discussion section.

Reviewer 2

> 1. In the Author summary, the authors mentioned that the descriptions of biological mechanisms are "overly complicated" yet "insufficiently comprehensive and detailed". Could the authors explain the meaning of this? By which means the descriptions of biological mechanisms are overly complicated? And by which means the descriptions of biological mechanisms are insufficiently comprehensive and detailed? To my understanding, trying to make the cell cycle model comprehensive and detailed results in the complication.

A similar comment was also raised by reviewer 3, and we agree that the wording is indeed confusing. While it is true that comprehensiveness and detail correlate with complicated descriptions, not all descriptions of the same reality are equally complicated. For instance, several mathematical problems become easier to solve after transformation of the coordinate system. In this concrete case, we are referring to the circumstance that rule-based descriptions of the cell cycle are more efficient, structured and less complicated than reaction-based descriptions or even bare-bones computer code. We tried to rephrase the sentence, but also have to keep the word limit of the Author summary in mind.

> 2. In line 123 at the G2/M transition submodel part, authors wrote "As B55 inactivity can stabilize its inactive state almost independently of CycB:Cdk1,...". In Figure 1e, CycB:Cdk1 will increase pGw, increase pEnsa, then increase pEnsa:B55. CycB:Cdk1 will cause the inactivation of B55, why B55's inactive state is independent of CycB:Cdk1? Or B55 inactivity can stabilize its inactive state almost independently of tCycB?

Thank you for this suggestion. We adapted it in our manuscript, which improves readability a lot. Nevertheless, to respond to your question on why B55's inactive state is maintained even when CycB:Cdk1 has fallen to very low levels, the likely reason is that inactive B55 cannot dephosphorylate pGw. So there is less CycB:Cdk1 needed to keep pGw phosphorylated.

> 3. In line 133 at the M/A transition submodel part, authors wrote "In contrast to CycB:Cdk1, pEnsa:B55 at the upper bifurcation point is much lower than the pEnsa:B55 at the lower bifurcation point (Fig. 1f), allowing to couple the bistable G2/M switch with B55 driven negative feedback (Fig. 1g)". In Fig. 1f, what is the approximate value of pEnsa:B55 at the upper bifurcation point and at the lower bifurcation point? What is the approximate value of CycB:Cdk1 at the upper bifurcation point and at the lower bifurcation point? I saw that the difference between pEnsa:B55 is at around 0.2 between the two bifurcation points, and the difference between CycB:Cdk1 is about 0.7. What does the pEnsa:B55 "much lower" mean?

The values for CycB:Cdk1 at the upper bifurcation point and lower bifurcation point are 0.110 and 0.068, respectively. The values for pEnsa:B55 at the upper and lower bifurcation point are 0.112 and 0.248, respectively. We have added these to the manuscript, apologies for the confusion.

> What is the meaning of "..., allowing to couple the bistable G2/M switch with B55 driven negative feedback (Fig. 1g)"? The much lower of pEnsa:B55 at the upper bifurcation point allow the limit cycle exist after coupling the bistable G2/M switch with B55 driven negative back? Could the authors give some explanations of the importance of pEnsa:B55 at the upper bifurcation point on coupling G2/M and MA? What will happen if the "much lower" doesn't exist?

Thank you very much for this question. We read the text again and realized that we omitted major steps in our reasoning and added them to the manuscript. To expand further on this explanation, the idea is that, if we want to degrade cyclin B, we need to phosphorylate Apc. One way to do this is by increasing the concentration of the Apc kinase CycB:Cdk1. The other is to decrease the concentration of an Apc phosphatase. Let us assume a purely kinase-driven mechanism, i.e. dephosphorylation of

Apc is constant. Let us further assume quick equilibration of pApc:Cdc20. Then pApc:Cdc20 amount monotonously increases with CycB:Cdk1. Using the (strongly idealized) mental model of a typical relaxation oscillator, what we would have to do to get oscillations is to find a cyclin B synthesis rate that is (1) high enough to push CycB:Cdk1 above the upper bifurcation point at 0.110 AU and (2) low enough that to allow CycB:Cdk1 to be degraded below the lower bifurcation point at 0.068. Even if pApc:Cdc20 were constant, no such synthesis rate could be found, because 0.110 is greater than 0.068 (and the situation is even worse, since pApc is monotonously increasing with pApc:Cdc20). However, considering the location of the bifurcation points for pEnsa:B55, we don't run into this issue if we now use a purely phosphatase driven mechanism.

> 4. In line 137 at the M/A transition submodel part, authors wrote "Combining the negative feedback.... results in limit cycle oscillations...". I think that the dCycB:Cdk1/dt equation (17) will become the dCycB:Cdk1/dt equation (26) in the supplemental file. Does this also mean that the saddle-node bifurcation in Fig. 1f of CycB:Cdk1 will become the Hopf bifurcation in Fig. 1h by change the Cyclin B synthesis rate (ksyCb in equation (26)) at the supplemental file?

Yes, the dCycB:Cdk1/dt equation (17) will become the dCycB:Cdk1/dt equation (26) in the supplemental file. Additionally, tCycB becomes a variable. It can therefore no longer be used as bifurcation parameter. Instead kSyCb is the bifurcation parameter. Since the bifurcation parameters differ, we do not think we can compare bifurcation points directly. However, it is true that there are no stable steady states and saddle-node bifurcations after combining the negative feedback from the MA transition submodel with the G2/M switch. Instead, we have stable limit cycle oscillations and Hopf bifurcations.

> 5. In line 145, author wrote "Without changing parameters, we could further demonstrate that the model also agrees with knockout experiment of CycE [38, 39] or CycA [40]." As there still is a stably oscillating cell cycle, does this mean that cells can still finish the cell cycle after CycE or CycA knockout?

Yes, they actually show continued proliferation. We clarified that in the manuscript.

> 6. After fused the four submodels to stably oscillating cell cycle model, I believe that there are two cell cycles shown in Fig. 2a, b. It will be helpful if the authors can label the start and end of each cell cycle on the top of the plot. Is the Hopf bifurcation still exist after fusing the four submodels? Is the Hopf bifurcation in Fig. 1h still exist by changing the bifurcation parameter CyclinB synthesis rate after fusing the four submodels? If the Hopf bifurcation still exist, the bifurcation parameter CyclinB synthesis rate range and amplitude of the limit cycle will be different?

Thank you for the suggestion to add labels for the start of the cell cycle. Since finding the precise start location is challenging on the time course plots, we decided to indicate the location of the M/A transition. This should still make it easier for the reader to count the number of cell cycles shown. Regarding the Hopf bifurcations, these still exist with respect to several parameters. For instance, we show in Fig. 2c,d that there is a choice of CycD (a parameter, not a state variable in our model) for which the model oscillates and a choice for which is does not. The particular parameter for the synthesis rate for cyclin B does no longer exist in the full model. This is because it was replaced with a baseline synthesis rate `kSyCb1` and a FOXO driven synthesis rate `kSyCb2`. We tried setting either of them to zero and observed that oscillations stopped when `kSyCb2 = 0`, indicating the existence of a Hopf bifurcation for `kSyCb2`. During the model fusion process, we had to add new species, add/replace reactions and adjust parameters. Therefore, it is expected that parameter ranges for Hopf bifurcations and amplitudes of cell cycle regulators do change compared to the M/A transition submodel. For instance the amplitude for CycB:Cdk1 is larger in the full model (Fig. 2), compared to the G2/M–M/A submodel (Fig. 1h and stable cycles in Fig S2).

> The orange line represents the CycB:Cdk1 and its peak is about 0.6 then decrease, during this 400 to 500 min, does the CycB:Cdk1 reaches a limit cycle? Because the limit cycle amplitude shown in Fig. 1h is about 0.2, I am not sure that if the Hopf bifurcation is still exist after fusing the four submodels.

In the fused model, the time between 400 and 500 min is only a small part of the limit cycle trajectory. The full limit cycle trajectory comprises the full cell cycle. In Fig. 2, we show two or more cycles. The initial conditions of the first cycle don't lie exactly on the limit cycle trajectory, but after approximately one cell cycle, the system approaches the limit cycle trajectory very closely. As stated in the response to the previous question, there exist Hopf bifurcations for several parameters (but the limit cycle trajectory looks different. It even enters dimensions that were not existent in the G2/M–M/A submodel).

> 7. To my understanding, the stably oscillating is most important characteristic after fusing these four submodels. There are experimental evidences showing the existence G1/S transitions and G2/M transitions. But methodologically, could the authors give some explanations on the necessity of the toggle switch of the G2/M submodel, Hopf bifurcation after combining the negative feedback of M/A transition, or the bifurcation in G1/S for the stably oscillating after fusing these four submodels?

The reason we created the four submodels prior to creating the full model is twofold. First, we wanted to break down the task of building a full cell cycle model into less complex sub-tasks. To asses if the sub-tasks were completed successfully, we checked if we see the expected bifurcations. Second, after fusing submodels, we wanted to obtain a stable limit cycle trajectory that can be turned into the stable steady states that describe several arrest states by changing parameters like CycD and TP53 accordingly.

After merging the submodels to the full model (which included adding a few more species and reactions, and adjusting the parameters to obtain oscillations (even under CycE and CycA knockout) and a restriction point), we obtain a dynamic system with a stable limit cycle. But there are easier ways to create an oscillating dynamical system that mimics the cell cycle. So it may seem that the bifurcations introduced by the submodels were unnecessary.

However, stable oscillations are not the only important characteristic we wanted to describe with our model. We also wanted to capture perturbations. And indeed, when lowering the CycD parameter, there is a Hopf bifurcation that turns the stable limit cycle into a stable steady state. This stable steady state roughly corresponds to the low CycE stable steady state of the restriction point submodel.

Similarly, when we introduced a DNA damage checkpoint in model version `v3.1.0`, there is a Hopf bifurcation that turns the stable limit cycle into two steady states when increasing the TP53 parameter. One steady state roughly corresponds to the low CycA steady state in the G1/S transition, the other roughly corresponds to the low CycB:Cdk1 steady state.

> 8. In Fig. 2a,b,c,d, is the stably oscillating independent of the initial values of the state variables? For example, if CycD = 1, the ODE simulation will be stably oscillating no matter of the initial values and there is no stably oscillating no matter of the initial values if CycD = 0?

It depends. The model contains several mass conservation relationships. For instance, the initial condition of Apc is set to 5, the initial conditions of all other Apc containing species is set to zero, and there is no Apc synthesis and degradation in the model. Changing the initial conditions without preserving total Apc = 5 can therefore change whether the model can oscillate or not. However, for the species Ca, Cb, Cdc20, Ce, E2f, Emi and Fox, we have written explicit synthesis and degradation reactions. To address your interesting question, we ran simulations with multiple random initializations for these species. We always observed oscillatory behaviour for CycD = 1 and never observed oscillatory behaviour for CycD = 0.

> 9. In line 170, could the authors give some explanations on “the re-entry trajectory does not follow the same path as the exit trajectory”? Please point out the simulations at which time periods in Fig. 3 are not the same?

Thank you for bringing this up. We tried to improve our descriptions in the manuscript, while also staying concise. Additionally, we added an example that should make it more clear how our model also shows different exit and entry trajectories. For a more detailed discussion on that matter, we would like to point the reader to the cited paper by Stallaert *et al*.

> 10. What does the cell density p mean? Is it the density of the cell number? In line 192, could the authors give some explanation on equation (1)? Will r be calculated from the imaging data in order to calculate the t? What does r mean in the imaging data and how to calculate it?

p(t) is a probability density function describing the probability that a cell chosen from an asynchronously dividing cell population had its last cell division event t hours ago. Your interpretation of r and t is correct, although we have to admit that it was not clear from the manuscript, so thank you for bringing this to our attention. We tried to clarify the concept in the text before we start with the derivation of Equation 1. Essentially, r describes the rank of a cell in a cell population that is sorted based on how far the cells have travelled along a reconstructed trajectory since cell division. The approaches for reconstructing the trajectories and ranking the cells differ from algorithm to algorithm. The basic idea of reCAT is to cluster the cell population in e.g. 8 groups, based on RNA or protein concentrations, using Gaussian mixture models. Next, reCAT tries to find the shortest path to visit all cluster centers (i.e. solve a travelling salesmen problem) to obtain a cell cycle trajectory along which they can rank the cells. As suggested by reviewer 3, we also added a schematic figure to illustrate the trajectory reconstruction algorithm. For a more in depth version, we encourage interested readers to refer to the cited publications.

> 11. In line 195, what is the meaning of “cell density decreases exponentially over the cell cycle with one cell cycle length half-life.”?

We agree that this was not well described and now point to the right equation in the manuscript to clarify the meaning.

> 12. Duration the parameter estimation, are the initial values of the model variable treated as parameters and estimated? Or the initial values are fixed?

During the parameter estimation, we also estimated the initial values of the model variables, as described in the caption of Fig. 5. More generally, which parameters are estimated and which are not is indicated in the ‘estimate‘ column of the PEtab parameter table in the linked GitHub repository.

> 13. In line 258, authors wrote “. . . were rescaled to a mean of one.” I saw that the protein values in Fig. 6b, d don’t have a mean of one, what does “. . . rescaled to a mean of one’ mean?

Well spotted, thanks! The nuclear species were rescaled to a mean of one and the cytoplasmic species were rescaled to preserve the nuclear/cytoplasmic ratio. To avoid confusion, we now provide an alternative reason to motivate a formula mapping observables to species in the revised manuscript.

> 14. In line 374, does the variable v mean a state variable in the model? Could the authors give some explanations on the equation v tilde?

Yes, v is a state variable. Originally, the reCAT algorithm was used to reconstruct cell cycle trajectories from transcriptome data, which was several order of magnitudes higher than the values (in arbitrary

units) from our simulated data. Empirically, we found that rescaling the state variables has a slight effect on the performance of the algorithm. Using a mean of approximately 10^4 lead to excellent correlation of reconstructed and ground truth cell ordering. The addition of 1 and taking the log_2 is identical to the transformation performed in the original publication. When feeding the immunofluorescence data from Stallaert *et al.* to reCAT, we took care that we transform to a similar order of magnitude as we have done with the simulated data. We tried to incorporate this response into the revised manuscript.

> 15. After the parameter estimation the imaging data, these parameters can obtain sustained oscillations and fit the data. What about the bistability of the submodel? I saw the model are modified based on Supplementary Note 1: Merging submodels, if these modifications are omitted after optimization on the imaging data, will the submodels still have the bistability show in Fig. 1 based on the estimated parameters? To my understanding, during the parameter procedure, the optimization algorithm doesn't guarantee the existence of the bistability for each submodel and the submodel could lose its bistability with the estimated parameters? If optimization procedure doesn't guarantee the existence of the bistability in submodels, similar to my question 7, what is the necessity of the bistability in the submodels when used the full model to fit the experimental data? Are the sustained oscillations the only necessity when trying to fit the experimental data because the bistability in submodel could not be exist after the optimization?

This is a very interesting question. Thank you for bringing it up. The G2/M transition submodel provides a great framework to think about it. In Fig. S3a, we see that there are two different stable steady states in the pEnsa vs. CycB:Cdk1 plane. However, if we allow the system to exit this plane into the pApc:Cdc20 direction, we see that we obtain a stable limit cycle or a stable steady state, depending on the parametrization (Fig. 1h and Fig. S4).

But indeed, the parameter fitting procedure only enforces behaviour seen in the data for fitting. We only used data for oscillatory behavior. Therefore, the parameter fitting procedure did not enforce the cell cycle arrest states for low CCND and high TP53. Building in these bistability/arrest states just makes our model more future-proof in the sense that it should be fittable to various perturbation conditions/arrest states once a suitable dataset becomes available.

> 16. There is a 0 to the power of 3 in Table 1 Observables column Gauthier et Pohl (2011). What does this mean?

We decided to use numeric indexing for the 9 footnotes in the table. We are aware that this is somewhat confusing, but perceived that as less confusing than indexing with various pairwise combinations of e.g. #, * and @.

> 17. Could the authors provide a Parameters Table similar to the Table 1 at the supplemental file?

Thank you, it seems that we did not clearly communicate how the parameters can be found, since reviewer 3 had similar issues. Due to the large number of model versions and to facilitate both, human and machine readability, we provide all parameter tables online on GitHub and Zenodo. To increase findability, we added links to the figure captions in the manuscript. For instance, the parameter table for the optimized model verb—v4.0.0— can be found in the
versions/v4.0.0/results_20230414/parameters_v4.0.0_optimized.tsv file of the cell_cycle_petab GitHub directory.

> 18. In the Fig. 3 at the supplemental file, pWee1, Cdc25 and Gw are explained in the captions, but they are not shown in the plots.

Thanks a lot for catching this mistake. These variables are only relevant in the caption of the reaction network (Fig. 1e). We removed them from the caption of the figure in the supplementary file.

> 19. What is the criteria used in classified G0 cells shown in Fig. 9 at the supplemental file? Are there xaxis and yaxis in the plot?

PHATE is a dimensionality reduction method, so the axis labels of the 2-dimensional PHATE plot could simply be called PHATE1 and PHATE2. We decided to omit plotting the figure axis and their labels as the labels (PHATE1 and PHATE2) would not be very informative and the axis were not visually appealing in combination with the colorbar. However, we modified the caption to make this clear. Regarding the classification of G0 cells, Stallaert *et al.* have shown that the central and right arm are part of the proliferative trajectory, whereas the left arm corresponds to G0 cells. The red line was manually chosen to separate the left arm from the central and right arms. This too is now clarified in the figure caption.

Reviewer 3

Main comments/questions:

1. Model parameters.

> - I did not find any table of used model parameters? This would be good to add, certainly for the simulations shown in Figs. 1-2. As the number of parameters (e.g. mentioned in Table 1) is very large, in particular for model 4.0.0, I understand it is difficult to put all parameters here in a Table. However, perhaps the most important ones and/or the ones that are experimentally confirmed can be added (see next point)?

Thank you, it seems that we did not clearly communicate how the parameters can be found, since reviewer 2 raised a similar concern in point 17. Please refer to our response there.

> - How were the model parameters chosen? Some reasoning is explained in the text, but which parameters are chosen based on experimental measurements of rate constants / concentrations / etc., and which are more arbitrary? This would be good to mention (in the table), or only show the experimentally motivated parameters.

Prior to parameter estimation, all model parameters were chosen manually (except for kDipEB55 and kAspEB55 in the G2/M model from Vinod et Novak, see also the revised section "Developing the Core model" in the methods). The parameters were selected to show that there is a choice of parameters that results in the desired behaviour of the reaction network (i.e. bistability for the RP, G1/S and G2M submodels, oscillations for the G2M/MA submodel, oscillations and cyclin D dependent restriction point in all unfitted model versions from `1.0.0` onwards. For `v3.1.0`, we additionally required a TP53 triggered G1 and G2 DNA damage checkpoint.). Concentrations are in arbitrary units.

> - Which parameters do you find from the optimization, both starting from the synthetic data (mimicking experimental data) and the actual experimental data? Are the estimated parameters logical, e.g. do they correspond to experimentally measured rates? Do we learn something new from the estimated parameters in terms of how the network functions dynamically?

Thank you for bringing this up. We indicated which parameters are estimated and which are fixed in the figure captions of the respective optimization problems. The estimation results for the synthetic data are logical (i.e. match the ground truth in principle, as explained in the manuscript). However, the question on which estimation results for the actual experimental data are logical is difficult to address for several reasons. First, there are barely any experimental rates known. Second, they often do not map well to our model rates (e.g. our kSy* rates are an agglomeration of transcription and translation rates). Third, we do not know how rates differ between our cell line (RPE1) and the cell lines for which data is available. However, the biggest problem is that most optimization problems in systems biology suffer from structural identifiability; and ours are not an exception. Structural unidentifiability means that the globally optimal solution is not a point, but a manifold (think of a line/curve in 2D space or a (hyper)surface in higher dimensional space) of infinitely many points. Additionally, we are not aware of methods that efficiently determine which parameters are identifiable (and thus biologically meaningful) and which are not for models of this size. Due to these limitations of typical systems biology optimization problems, we believe that there is not much point in comparing estimated parameters to direct experimental measurements. It is therefore not surprising, that the only two experimentally determined parameters of our model we are aware of ($kDipEB55$ and $kAspEB55$) differ substantially from their experimentally determined values after fitting model version `v4.0.0`. Our best bet is that this is due to structural unidentifiability. After all, we don't observe the pEnsa:B55 complex. In the discussion section we explain that genetic and small molecule perturbation experiments, combined with time course measurements are needed to mitigate this issue. Additionally, we added a short statement on the unidentifiability problem in the results section.

> - For the perfect synthetic data, you show that the ground truth can be recovered, so you find the global minimum. For the rescaled synthetic data and the experimental data, this is not discussed. Could you say something more on how often you find different local optima, whether those perhaps approximate the data very well too, and how they differ in their parameter profiles?

We realized that we were too concise when mentioning the unidentifiability problem in the results section. While a detailed discussion of parameter unidentifiability is not the scope of this manuscript, we added a short statement to discuss its major ramifications. Regarding local optima, we already found 100 different local optima with 100 multistarts for perfect simulated data, none of which approximated the data very well. This motivated us to use (saC)eSS, instead of multistart optimization. In section "Imitating real imaging data and speeding up optimisation", we explain why the imitation of real data leads to degeneration of the global optimum from a point to a whole manifold. The same argumentation also holds for the local optima. Similarly to the unidentifiability issue, the local vs. global optimum issue is caused by (mathematical) properties of the optimization problem with little biological relevance. We therefore did not analyze the parameter profiles in more depth.

> - Saying something more about the influence of sampling rate and noise amplitude on parameter estimation could be informative.

This is a very interesting comment in the context of the non-uniform sampling rate in our work. As explained in the manuscript, the sampling rate exponentially decreases by a factor of 2 over the whole cell cycle, meaning that the earliest cell cycle stages contribute twice as much to the loss function as the latest cell cycle stages. We could have corrected for that by weighting the datapoints accordingly, which is equivalent to modifying the variance of the measurement noise. Unfortunately, we are not aware of any literature that discusses this matter in light of probabilistically motivated objective functions. Intuitively, if all (even consecutive) datapoints of the time course are statistically independent, then no correction should be applied. Conversely, if two consecutive datapoints are drawn from the exact same distribution (i.e. the limit of infinite sampling frequency), then the correction should be fully applied. These two edge cases aside, it seems that in any realistic scenario a partial correction should be applied. However, since our sampling frequency only varies by a factor of two, it did not seem that correction of sampling frequency would have a strong effect on the result of the optimization. We therefore chose to stick to the simple approach of not correcting for sampling rate.

2. Model structure.

> - How does this extended cell cycle model differ from other cell cycle models? The authors compare some key aspects in Table 1, but I would find it interesting to expand the discussion on the novelty of the current model vs. such previous models in the main text. For example, how does this molecularly detailed model differ from others which also aim to incorporate molecular detailed interactions? Recently, De Boeck et al. (PloS CB 17, e1009008, 2021) also introduced a cell cycle oscillation model that incorporates the various bistable switches mentioned here. However, they omitted the molecular detail underlying each bistable curve, focusing only on the functional motifs, leading to much less parameters that need to be estimated.

We really believe that the novelty of our approach lies in the combination of the features mentioned in the discussion section. We think there are no major differences to other ODE models in how we aim to incorporate molecular detail. Perhaps one small difference is, that we use mass action kinetics in all rate laws (except for nuclear envelope breakdown, which we switched off during parameter estimation for performance reasons). We added this to the main text in the discussion.

Several reactions using mass action kinetics can be combined to ultrasensitive responses as requirement for bistability. As described by De Boeck and others, such ultrasensitive responses can also be achieved with more phenomenological descriptions, like Hill functions. In some sense, using a single Hill function (e.g. multi-site phosphorylation) reaction is a surrogate model for coupled (e.g. single-site phosphorylation) reactions. Similarly, using mass action kinetics and a well-mixed assumption can be viewed

as surrogate model for agent-based 3D models of molecule collisions. What mechanistic detail can and should be replaced with surrogate models depends on the questions that shall be addressed with the model. It is great to see that De Boeck *et al.* introduce surrogates for more complex cell cycle reaction motifs and fuse them to an oscillating cell cycle model. This warrants recognition in our manuscript. If systems biologist ever want to build models of whole cells, such surrogates will likely be needed to simulate these models in a reasonable time frame.

> - (Related to point 1) If you fit the different model versions to experimental data, to which extent do key model parameters stay the same or change significantly? As the title includes ”compartment-resolved”, how do the model parameters change in the model version with/without compartments. If the estimated parameters change with every model version, I wonder whether this is a problem? As new interactions are also still being discovered, I can imagine that one can never really capture all molecular details, so perhaps one wants to see that a large part of the parameters remains unchanged across model versions.

Due to parameter unidentifiability, even if we were to perform two optimization runs on the same model, we expect to obtain different parameter vectors. Therefore, we have not compared parameter vectors of different models. Of course, adding more detail will change the model, the dimensionality of the parameter landscape and the location of the optimal parameter manifold (even after projection into a comparable lower dimensional subspace). Therefore, re-optimization is required. However, the parameter values of smaller models can be used as initial guesses for fitting the parameters of larger models, based on the assumption that this guess is better than random and will therefore speed up the optimization run.

Now is it a problem that the parameters change after every optimization, i.e. is unidentifiability a problem? We think it depends. If one purely intends to formalize existing biological knowledge into an executable model to check if the existing knowledge of the reaction mechanisms is consistent with experimentally observed time courses (i.e. check if there is a parametrization that fits the data well), then unidentifiability is not a problem. However, if the model shall predict future experiments, then unidentifiability is an issue. This is because unidentifiability results in loss of predictive power for some *in silico* experiments. This problem can be managed by running the optimization several times to find an ensemble of different parameter vectors with identical objective function value. *In silico* experiments can then be run with all parameter vectors. Only if the simulation result for the observables of interest are the same for all parameter vectors, the unidentifiability did not effect the predictive power for this particular in silico experiment. This is expected if the *in silico* experiment and observables are sufficiently close to the experiment and observables the model was trained on.

3. Time-course reconstruction.

> To which extent does the reconstruction of a time course from snapshots of many different cells give a realistic picture of the behavior of an individual cell? Some short discussion and visualization (sketch) on how this reconstruction works would be useful. I guess this generates an averaged cell cycle trajectory? However, I expect individual cells to follow quite different trajectories, corresponding to (strong?) heterogeneities in the equivalent model parameters describing each cell (perhaps even time-dependent parameters in response to changing conditions).

Our goal was to create a model for the ”average” cell. Practical constraints aside, let’s assume we would have live cell imaging time courses of 300 cells for the same targets that were measured in snapshot 4i experiments. Due to (a) heterogeneities in the parameters and (b) molecular noise, these cells would move at different speed through different cell cycle phases. To generate an average trajectory, we would have to align all the time courses. For a single cell, this could mean that minute 61 to 62 post division needs to be stretched by a factor of 1.1, but minute 62 to 63 needs to be stretched by a factor of 0.9 and so on (assuming discretisation of time into minutes). But what does it mean for a cell to be in a certain phase? Of course, we know the 4 phases G1, S, G2 and M, which are defined by their molecular state, not by wall time. So we need to distinguish wall time and, to use the terminology introduced

by Stallaert *et al*, molecular cell cycle age. So it would be good, if we could align the trajectories to a canonical cell cycle age. And this is exactly what reCAT and other trajectory reconstruction methods are doing. It is important to note that for the reconstruction from snapshot data, one cell casts one vote for one time point, instead 1/300 vote for all time points when aligning live cell imaging trajectories. The latter would lead to averaging/smoothing of variabilities from (a) parameter heterogeneity and (b) molecular noise. The former draws independent samples of (a) parameter heterogeneity and (b) molecular noise. In Fig. 4, we used a Kalman filter to smoothen the time courses. For parameter fitting, the rigidity of the ODE model performs an implicit smoothing, accounting for the noise model that was part of the likelihood function and defined in PEtab. Taken together, the reconstruction of a time course from snapshots of many different cells differs from the behaviour of an individual cell, by averaging parametric heterogeneity and randomizing molecular noise, which was both desirable for the goal of fitting to an "average" cell. We provide a sketch of the reconstruction in Fig. 4b and mentioned in the text that we obtained an "average" trajectory in some sense.

> Does this lead to challenges for the reconstruction of the cell cycle time courses and model fitting? Later in the paper, synthetic data is generated from a model with fixed parameters in the case with/without noise on the variables/observables. What if noise on the parameter values is introduced?

Since we want to fit the model to the average cell, we are OK with heterogeneities when reconstructing the trajectory, as long as the cell culture is asynchronously dividing (which is why we excluded G0 cells). Regarding estimating the noise in parameter values, the probably most rigorous framework would be nonlinear mixed effects (NLME) models. Unfortunately, we are not aware of NLME approaches that handle parameter unidentifiability well, so that Quantitative Systems Pharmacology (QSP) modeling strategies might have to be chosen as viable alternative. While studying parametric heterogeneity in the context of cell cycle control is certainly a very interesting topic, our goal was to describe the "average" cell in a mathematical model. For this reason, we also did not introduce parametric uncertainty when generating synthetic data. However, introducing parametric heterogeneity would have better mimicked the sources of variability in the real dataset. Had we done this without reducing the noise on the observation, we would have seen more variability overall. Likely, some cells would have entered arrest states, so that we would have had to perform a similar cleaning step as we did for the real imaging data before performing a trajectory reconstruction with reCAT.

Minor comments/questions:

> a. In the summary, the authors write "the descriptions of biological mechanisms are often overly complicated yet insufficiently comprehensive and detailed." It seems contradictory that the model is overly complicated, yet not detailed enough? This is repeated in the introduction, but without the "detailed", the contrast between detail/complicatedness vs comprehensive/understandable does make sense.

Reviewer 2 had a similar comment in question 1. Please refer to our response there.

> b. L43: "tools have not yet been exploited to refine existing cell cycle models". This statement seems a bit too strong. Researchers have used those different elements to refine models, but rather not in the same way and to the same extent as here?

While we are not aware of mammalian cell cycle models that have used either PEtab, 4i data, trajectory reconstruction algorithms or parameter estimation algorithms, let alone used them in combination, other tools/formats (SBML, BioNetGen) have been used. Additionally, there may always be work we are unaware of. So we agree that this statement is too strong. We now say "have not yet been fully exploited",

> c. L78 (and other places): "tE2f" > it would improve readability to define this in the main text. This also goes for the other instances where a "total amount" is used. It would also be easier if this were written more explicitly in the figures, e.g. Fig. 1f: tCycB -> total amount/concentration of CycB.

Indeed, "tE2f" etc. come a bit out of the blue. We defined them in the main text and Fig. 1f to improve readability.

> d. L80-81: one refers to Fig. 1B here, but I do not see how this sentence relates to Fig. 1B other than that there is bistability.

Thanks! We can see how the association between text and figure is not clear and modified the manuscript accordingly.

> e. Fig. 1b and Fig. 1d: Why are you plotting negative values on the x-axis, which is not biologically/physically relevant? In Fig. 1b I understand as you might want to show the whole curve, although I would (also graphically) indicate that the negative value range is not physical. In Fig. 1b I also find it strange how the bottom branch terminates: why there in the middle of the graph (around (-0.2,0.05))?

It does indeed look strange that the lower branch terminates. The reason is that the steady state is lost if CycD is further reduced. We indicated that this is an unphysiological condition, since it happens at a negative CycD concentration. The original reason to extend the x-axis of Fig. 1d to minus 1 is for consistency with the corresponding Fig. S2b (frankly, I also made the mistake to plot Figs. 1-2 and S1-S4 with a proprietary tool and my license ran out, so I can no longer change that easily).

> f. L113-115: The authors seem to limit themselves here to recent studies specifically focusing on the PP2A:B55/Ensa/Greatwall pathway in the mammalian cell cycle, although I think it could be nice to mention several other experimental works here that show the existence of multiple bistable switches in the G2/M transition: ref [70], ref [71], Mochida et al. Curr Biol 26, 3361 (2016), and Kamenz et al. Curr Biol 31, 794 (2020).

Thanks for this suggestion. We modified the text and added the citations.

> g. In Fig. 1h and Fig. S4, the authors show that there are limit cycle oscillations in the system. However, it seems that the oscillations do not "go around" the whole S-shaped curve, so it does not look like a typical relaxation-like oscillator. Why is this the case and does this also happen in the optimized models later in the manuscript? Could the authors give some interpretation? Is this because there are multiple bistable switches (if so, other projections could be informative)?

Indeed, as shown in Fig S4a, the oscillations do not go around the whole S-shaped curve. We can explain this observation as follows. In an typical relaxation oscillator, we would see quick jumps between the steady states, and slow movements along (asymptotically approaching) the branches representing the steady state. Once the system crosses the unstable steady state, the next jump is triggered. In the M/A transition submodel, however, the pApc:Cdc20 mediated negative feedback kicks in quite quickly, too, i.e. before the system has a chance to approach the high CycB:Cdk1 steady state. Similarly, CycB synthesis is fast enough that the system moves almost parallel to the lower stable steady state, without ever reaching it. However, when starting at tCycB = CycB:Cdk1 = 0, we see in the figure below that the system stays close to the lower steady state for longer.

We do not think multiple bistable switches are involved in this phenomenon, as we have implemented only one source of ultrasensitivity (i.e. inhibitor ultrasensitivity between B55 and pEnsa). As we do not

use Michaelis-Menten kinetics, the positive feedback of CycB:Cdk1 on itself via Wee1 and Cdc25 lacks ultrasensitivity, and is therefore not a bistable switch.

Unfortunately, it is tricky for us to do bifurcation analysis on the full fitted model. We used XPP for submodel development due to its nice functionality for bifurcation analysis. However, XPP is much less suited than BioNetGen/SBML for large models. We therefore do not have an XPP version of the full fitted model. We do however have model `v1.0.0` in XPP and tried to perform the requested analysis on this unfitted model. Unfortunately, even `v1.0.0` is quite a large model, and XPP crashed when trying to perform the bifurcation analysis.

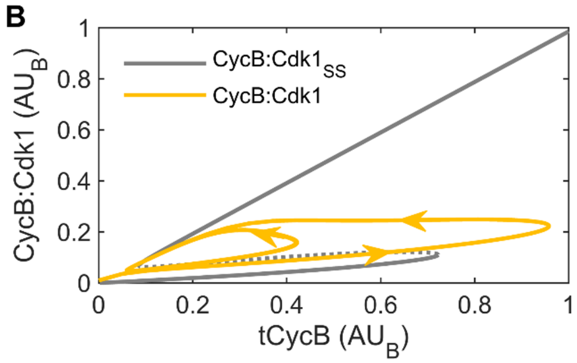

Figure 1 — M/A trajectory starting from tCycB = CycB:Cdk1 = 0

> h. L148: it could be useful to shortly mention the main changes here?

These changes are really minor, but to give the reader a better sense about the changes without having to go to the supplementary material, we listed two examples in the revised manuscript: changing initial conditions and rescaling of the time units. We chose those two examples among all listed points in the supplementary table as they are easy to understand. We think that some of the other points are harder to explain and may distract from the main story line, or even cause unnecessary confusion if mentioned in the main text.

> i. L149-151: why is extending the ODE-based model with additional interactions so difficult? This is not immediately clear to me. Is it just because having a visual, rule-based initial layer that automatically translates interaction pictures into ODEs is easier for the user and one is less likely to make a typo in equation terms? Is it true that eventually, using this BNG interface, one goes back to a system of coupled ODEs? In this sense one is not solving a different type of model, but it is just an easier user interface to get to the eventual ODE system? The BNG system also has the option to translate it to a Gillespie problem, which is useful, but not used here I think? I would find a short discussion to clarify these points useful.

Thanks for this feedback, we tried to add a concise clarification to the text. For further details, we would like to refer to Chapter 2.1 on Model Representation in Paul Lang's dissertation, as well as the literature cited there. Essentially, the rule-based initial layer is based on text (code), not graphics. The rules automatically expand to reaction a network. This reaction network can then be exported as SBML. SBML compliant software can then simulate this reaction system deterministically (after translation to an ODE system) or stochastically. The Rulebender software understands the BNG language, so that stochastic, deterministic (and even network-free) simulations are possible without SBML export.

The reason why extending the ODE-based model with additional interactions is so difficult boils down to human error. For instance, the intention of the modeller can be to introduce a new protein C that binds to an existing protein A. But perhaps A exists in the model in a phosphorylated or unphosphorylated form; and it might also exist free or in complex with a protein B; and B might exist in a phosphorylated

or unphosphorylated form, too, and maybe this phosphorylation is not relevant for the binding to A, etc. So instead of creating a single new differential equation for the A:C complex, the modeller has to create multiple new equations. Additionally, the modeller must modify multiple old differential equations. With complex models, this can quickly lead to human errors.

With a rule-based framework, the modeller just specifies the intention ”C binds to A, no matter what”. Finer granularity, such as ”C binds to unphosphorylated A, no matter what” is also possible. BNG then automatically expands this to a reaction network, avoiding human error. However, there is one obvious source of error left, namely that it could turn out that the expansion is different to what the modeller intended. Typically, the expansions may include reactions the modeller has not thought of or intended. To avoid this source of error, we made a note of the number of expected new species and reactions generated from each rule we added during model expansion, and compared that to the number of BNG-generated species and rules to resolve discrepancies (this note and the whole process is traceable on our GitHub repo).

We never ran the model with a stochastic simulation algorithm, since the noise level depends on the particle number, but we only use arbitrary units. So running with a stochastic simulation algorithm would not get the noise right, but it would take very long to simulate.

> j. L158-160: How did the BNG formulation facilitate the introduction of the DNA damage checkpoint, in which sense was this not possible (or much more difficult) in the regular ODE formulation? Please explain a bit more.

CDKN1A can bind to four different model states that were already present in the model prior to extension: cyclin E, cyclin A and (in)active cyclin B (we always implicitly assumed cyclins are bound to CDKs). CDKN1A can also be phosphorylated. But phoshpo-CDKN1A cannot bind to the cyclins. Now for the degradation of CDKN1A in an ODE based framework, we need to consider 6 different degradation reactions (one degradation reaction for its presence in each complex, plus one for free CDKN1A plus one for free phospho-CDKN1A). In the rule based framework, we would need just one rule: CDKNA1 is degraded regardless of binding of phorphorylation state. However, to specify that the phophorylated form is degraded at a different rate, we used two rules: one for CDKN1A in free or bound form, and another one (with a faster rate constant) for phospho-CDKN1A. In the ODE-based framework, we would also have to consider two association and dissociation reactions with cyclin B, one for active and one for inactive cyclin B. In the rule-based framework, this is just one rule for cyclin B in general. The same holds for phosphorylation mediated dissociation from (in)active cyclin B (there is no benefit of using the rule-based descriptions for phosphorylation mediated dissociation from cyclins E and A, as they have only one form). A similar argumentation holds for the addition of CDKN1B to the model. Additionally, when introducing the notion of compartments, we did not have to duplicate reactions in the rule-based framework. Instead, we simply specified that a rule acts regardless of the compartment, as long as the reactants are present in that compartment (with protein synthesis as notable exception, where we specified that the product ends up in the cytoplasm only).

> k. L196-199: A figure sketch as to how this method works with intermediate steps (sampling - adding noise - reconstruction) could be useful, even in a main figure. Similar remark when talking about mapping of observables. These are all important in handling the experimental data, so having it visually clear in a sketch could be nice for the reader.

We added a visualisation for sampling and reconstruction as a main figure. We show the noise-free case for better clarity. The case where we added noise is shown in Fig. S8. As the mapping of observables is difficult to illustrate visually, we added a clarifying description/example to complement the equation.

> l. L211-213: One talks about 36 features (out of 40), and then about 292 features. Where does the number 292 come from?

Apologies for the confusion. We tried to clarify this concisely in the text. Stallaert *et al.* labelled the cells with 48 different antibodies. Combined with structural analysis, this lead to 292 features for each cell

that were extracted from the microscopy image, i.e. a cell was represented by a vector of length 292. 40 of these features were identified by Stallaert as features that provide cell cycle information. 36 of these features do not contain missing values, which are not allowed in reCAT. We then sorted the cells (vectors of length 292) along the cell cycle trajectory with reCAT, based on these 36 features.

> m. Fig 5: The caption is hard to entirely understand as e.g. θ_{true} and eps are not defined/discussed in the main text.

Thank you for this feedback. We tried to improve the caption accordingly. We did not define $\boldsymbol{\theta}_{true}$ in the main text, as we believe axis labels are best explained in the caption.
