# Supplementary material for: Reusable rule-based cell cycle model explains compartment-resolved dynamics of 16 observables in RPE-1 cells
Source: PLoS Comput Biol. 2024 Jan 8;20(1):e1011151. doi: 10.1371/journal.pcbi.1011151 (PMC10773963; doi:10.1371/journal.pcbi.1011151)
Supplement: Attachment — Submitted filename: response_to_reviewers_cell_cycle_plos_2.pdf [file pcbi.1011151.s003.pdf]

November 5, 2023

Paul F Lang
Email: plang@biosim.ai

Frohburgstrasse 106
8057 Zurich
Switzerland

Dear Dr Chen and Dr Mendes,

Thank you very much for your continued interest in our manuscript. I am pleased to learn that the reviewers found the topic of our research important and that the manuscript is likely to be accepted for publication, pending minor revisions. I want to extend my appreciation to Reviewer 2 for their thorough review that helped us to enhance the quality and clarity of our manuscript.

In the following, you will again find our detailed responses to their comments. We have also included the revised manuscript with and without highlighted changes, and corrected a typo in the supporting information.

Sincerely yours,

Paul F Lang

Reviewer 2

> 1. In line 140, the author wrote "introducing a spindle assembly checkpoint that keeps pApc:Cdc20 inactive during the process of cyclin B:Cdk1 activation/phosphorylation, thus decoupling cyclin B:Cdk1 activation/phosphorylation from cyclin B degradation." Does this represent the equation (25) in Supporting Equations 4 that pApc:Cdc20 only degrades tCycB but CycB:Cdk1 complex? Is this modification biologically reasonable? It seems that Cdk1 protects CycB from the degradation caused by pApc:Cdc20.

We see how our terminology causes confusion. Thank you for bringing this up. It would indeed not be biologically reasonable to assume that CDK1 protects cyclin B from APC mediated degradation. However, in Table S1 we defined tCycB as "cyclin B:(p)Cdk1 complex", i.e. regardless of Cdk1 activation/phosphorylation. When Cdk1 becomes activated, we explicitly use it in the species name. That is CycB:Cdk1, is defined as "cyclin B in complex with unphosphorylated (i.e. active) Cdk1". Since the naming of these two species is confusing, we tried to clarify these on first occurrence in the main text.

> 2. A following-up question to the previous question 5, so the "continued cell proliferation" is considered as the existence of sustained oscillations of proteins, e.g., CycE, CycA and CycB:Cdk1. The other characteristics as period and amplitude of the limit cycle are not considered?

Thank you very much for this comment. The cited studies indeed contained information of period and amplitude for the limit cycle. Geng *et al.* found increased doubling time under CycE knockout, and Kalaszczynska *et al.* found elevated CycE levels and unchanged doubling time under CycA knockout. Both were captured by our model, and we adapted the main text accordingly. Additionally, expanded the main text to better explain the presence of a mitogen-depletion triggered restriction point in the full model.

> 3. In addition to the Supporting Note 5, will the removal of G0 cells from an asynchronous cell population help the parameter fitting procedure? Like the question 15, as the authors said, the parameter fitting procedure only enforces behavior seen in the data for fitting. But the optimization doesn't enforce the oscillatory behavior by itself. Is this another reason only used data for oscillatory behavior because there is no oscillatory behavior in G0 cells, and using the G0 cells will undermine the optimization process?

Thank you for following up. Let us first clarify that parameter estimation and time course reconstruction are two completely distinct procedures. For parameter estimation, we require time course data and corresponding experimental condition as input; for instance a time course of mitogen induced cell proliferation from cell division to cell division; or a time course of mitogen depletion-induced G0 arrest from cell division to a terminal G0 state. The more time courses for different experimental conditions, the better. Had we trained our parameters on a mitogen induced proliferative time course and a mitogen depleted G0 arrest time course, both behaviors would have been enforced in the fitting.

Unfortunately, however, it is to our knowledge not possible to reconstruct time courses for the G0 arrest trajectory from the 4i data. This is because we cannot apply Equation 2 to calculate how fast cells are moving along different stretches on the G0 arrest trajectory, as the underlying assumptions explained in the main text do not hold. However, Equation 2 can be applied to calculate how fast cells move along the proliferative trajectory; and to obtain a cell population that moves along this proliferative trajectory, we had to remove G0 cells.

More generally, it is important to emphasize that a key benefit of mechanistic (e.g. ODE) models is that they enforce certain behaviour in their equations. This makes them less data-hungry than machine learning models and improves the predictive power of extrapolation beyond the experimental conditions/data used for parameter estimation. On the other hand, parameter estimation for neural network models can

enforce anything, from describing time courses of biochemical reactions to classifying images, as long as sufficient data is provided.

> 4. In the table 2 of the Supporting Information, there are changes of 2.1.0 and 3.0.1 about the transcription factor E2f. What is necessity using E2f:PX as the transcription factor instead of simply using the free unphosphorylated E2f as the transcription factor? Will using E2f bound to the promoter as the transcription factor increase the robustness of the RP or G1/S transition in the submodel?

These changes are very minor and were introduced to better reflect biological knowledge. We did not experience any effects on the robustness of the RP or any other transitions. The change leading to `2.1.0` makes sure that free E2F is consumed upon promoter binding (despite the concentration of promoter-bound E2F is negligible). The change leading to `2.1.1` replaces the generic E2F activated promoter Px, from which all E2F-activated genes were transcribed, to gene-specific promoters. This is of course closer to the biological reality. Finally, the first point in the change leading to `3.0.1` was to ensure that not only unphosphorylated E2F, but also phosphorylated E2F can bind to the promoters. This change was introduced based on the assumption that E2F phosphorylation only affects E2F degradation, but not promoter binding.

> 5. In Supporting Note 6, what is modification to account for "simulations with these optimised parameters unexpectedly showed small dampened oscillations of observables for a short period of time." I think that the modification would be the introduce of Ok. But in line 300, authors wrote "Ok was set to zero for all k". Setting Ok to zero is only applied at Imitating real imaging data but not at Parameter estimation with imaging data?

Yes, your interpretation is correct. When fitting to imitated imaging data, we used zero offset, and when we fitted to actual imaging data, we realised that we need to lift this constraint to get good fits. We tried to make this more clear in the manuscript.
